# Immune modulating effects of continuous bioaerosol and terpene exposure over three years among sawmill workers in Norway

**DOI:** 10.5271/sjweh.4240

**Published:** 2025-09-01

**Authors:** Anne Straumfors, Fred Haugen, Øivind Skare, Wijnand Eduard, Paul K Henneberger, Jeroen Douwes, Bente Ulvestad, Karl-Christian Nordby

**Affiliations:** 1 National Institute of Occupational Health (STAMI), Oslo, Norway.; 2 National Institute for Occupational Safety and Health, CDC, Morgantown, West Virginia, USA.; 3 Centre for Public Health Research, Massey University, Wellington, New Zealand.

**Keywords:** fungal spore, IL-1β, IL-10, leucocyte, longitudinal, resin acid, serum biomarker, TNF-α, wood dust

## Abstract

**Objectives:**

Exposure to wood dust, resin acids, microbial and volatile components among sawmill workers may impair respiratory health, with inflammation indicated as a key mechanism. Previous, mostly cross-sectional studies have shown mixed results, and a conclusive association between wood dust exposure and chronic respiratory inflammation has therefore not yet been established. This study assessed associations between exposure to bioaerosols and volatile terpenes and serum inflammatory marker levels over three years.

**Methods:**

Serum biomarkers and blood cell counts were analyzed based on 702 observations from 450 exposed sawmill workers and 102 observations from 65 unexposed sawmill workers in Norway at baseline and after three years. Job-exposure-matrices, based on measurements among the same cohort, were used to assess exposures for wood dust, endotoxins, resin acid, monoterpenes, fungal spores, and fungal fragments. Changes in exposures, biomarkers and cell counts over the study period, as well as group differences and potential cause-and-effect associations were assessed using linear mixed regression.

**Results:**

Exposures were relatively low and below occupational limits, although variances were relatively high (GSD_tot_ 2.1–8.3), largely driven by differences between workers (GSD_bw_ 1.9–7.8). Serum CC-16 and mCRP were slightly higher after three years, whereas IL-1β, TNF-α and IL-10 levels were significantly lower among exposed compared with unexposed workers. Exposures positively associated with increases in biomarker levels included endotoxin with mCRP, monoterpenes with IL-10, and fungal spores with TNF-α and IL-8. Exposed workers had higher counts of total leucocytes, neutrophils, lymphocytes and basophils after three years. Several of the increased leucocyte counts were associated with concurrent increase in mCRP and IL-6 concentrations, predominantly in the exposed group. Conversely, increased CC-16 levels were associated with lower leucocyte and neutrophil counts, mainly in the unexposed group.

**Conclusion:**

Continuous exposure to wood dust and related components for three years appears to induce a chronic low-grade inflammatory response among sawmill workers with a shift in cytokine profiles towards a less regulated, potentially more muted immune state.

Sawmill workers are exposed to wood dust, microorganisms, resin acids, endotoxins, and vapors containing terpenes (1), which are associated with significant health risks, including respiratory symptoms such as cough, wheezing, and shortness of breath (2–4). Pine dust has also been associated with skin irritation, allergy and respiratory symptoms including asthma symptoms and lung function decline (5–7). Moreover, chronic exposure to dust from both hard and soft wood may cause nasal and sinonasal cancer (8) and possibly lung cancer (9, 10), although endotoxin exposure may reduce the risk of lung cancer (11). While the mechanisms for the latter are not fully understood, immune stimulation and modulation may play a role (11, 12). In particular, the lipid A portion of endotoxin has been shown to suppress tumor growth in animal models (12), but it also elicits inflammation and is responsible for both innate immune recognition and control of adaptive immune responses, depending on its structural acyl variations (13). Additionally, wood dust-associated resin acid, terpenes and microorganisms may modify the inflammatory response to wood dust exposure (1, 14–16). Despite this, and the known health risks, the effects of long-term wood dust exposure on inflammation and resultant chronic respiratory disease are not fully understood.

Previous, mostly cross-sectional, studies on wood dust and inflammation have shown mixed results, and many knowledge gaps remain. Softwood induces the production of pro-inflammatory cytokines (interleukin-6 (IL-6) and IL-8) in human epithelial cells, and pine wood also induces DNA strand breaks (17). Mouse studies have shown that repeated airway exposure to wood dust can elicit lung inflammation, with elevated levels of proinflammatory cytokines [IL-1β, tumor necrosis factor-alpha (TNF-α)] and chemokines [including C-C motif chemokine ligand 8 (CCL8)] (18). To date, few studies have considered a wide range of inflammatory markers and multiple exposure components simultaneously, limiting the understanding of sawmill-dust-associated immune responses.

Several inflammatory markers may play a role. TNF-α is a key pro-inflammatory cytokine involved in the response to microbial infections and environmental exposures. High fungal exposure has previously been reported in sawmills (19), which has the potential to activate the immune system through pattern-recognition receptors, leading to the recruitment and activation of immune cells (20, 21). In particular, activation of Toll-like receptor (TLR) initiates signaling cascades that lead to the activation of transcription factors like nuclear factor kappa B (NF-kB) and activator protein 1 (AP-1), which drive the expression of pro-inflammatory cytokines, including TNF-α (22, 23). TNF-α can also enhance the TLR signaling through a feedback loop that amplifies inflammatory responses either through increased TLR expression or by boosting the production of other pro-inflammatory cytokines like IL1-β, IL-6 and IL-12 (24). While TLR trigger TNF-α production as a protective response, excessive or prolonged activation may lead to chronic inflammatory diseases. IL-8 is a widely studied chemokine and a critical inflammatory mediator, which mobilizes leucocytes to sites of inflammation (25), and serum-levels are often elevated in workers exposed to high levels of bioaerosols (26–28). Changes in serum levels of pneumoproteins, such as the anti-inflammatory club-cell protein 16 (CC-16) or surfactant protein A and D (SP-A and SP-D) may indicate lung injury following inhalation exposure to dust (29, 30). As an anti-inflammatory cytokine, IL-10 helps to regulate the immune response and prevent excessive inflammation (31, 32).

This study examined associations between serum levels of specific inflammatory markers, blood leucocyte counts, and exposure estimates of wood dust, resin acid, microbial and volatile components over a three-year period in a cohort of sawmill workers. We hypothesized that exposures experienced by workers in contemporary sawmills causes an inflammatory response reflected by increased levels of cytokines and pneumoproteins, with the expectation that improved insights into these inflammatory responses may be useful in identifying early disease markers.

## Methods

### Ethical considerations

The Regional Committee for Medical and Health Research Ethics (REK #2012/1248) and the Norwegian Data Inspectorate approved this study. All participants gave written informed consent.

### Study population and study design

Eleven industrial sawmills, sorting, and planing companies in Norway, processing predominantly Norway spruce (*Picea abies*) and/or Scots pine (*Pinus sylvestris*) were included. Seven of the companies had sawmill, sorting, and planing departments, whereas four did not have a planing department, and one company had only a planing department. Companies were selected based on size, location, and wood type. To obtain a representative selection of companies, we recruited large- and medium-sized industrial plants from the two largest companies in the Norwegian wood industry and from independent sawmill companies. Small private sawmills connected to farms were not included. All employees were invited at baseline (winter of 2012/2013) and after three years (winter of 2015/2016). Exposed workers were defined as production workers exposed to wood dust (working with timber inside or outside the production facilities). Unexposed workers were administrative personnel working at offices, separated from the exposed production areas. Blood samples were collected from the workers in winter at both baseline and after three years, accompanied by the administration of questionnaires addressing smoking habits and health parameters.

### Job exposure matrices

Personal airborne exposure to wood dust, resin acid, monoterpenes and microbial components were measured during the winter and summer seasons in the period 2013–2014. In winter 2016, measurements of wood dust, resin acid and monoterpenes were repeated at one company that had built a new department for sorting dry wood, confirming that this change had no significant effect on already low exposure levels. This resulted in 1868 full-shift personal thoracic samples involving 1–6 repeated measurements of 205 workers nested within job types/departments and companies. The exposure results have been published previously (1, 33). Based on these measurements, job-exposure matrices (JEM) were developed to predict the exposure for each worker in year 1 and year 4, except for administrative workers who were considered unexposed. The exposure data followed log-normal distributions, and mixed-effect regressions of natural log-transformed exposure concentrations of thoracic dust, resin acid, endotoxins, fungal spores, fungal fragments, and monoterpenes, were used to predict exposure levels for workers, based on their job type/department and company. The regression analyses included company (N=1–11), job type/department (N=1–8), sampling year (2013, 2014 or 2016), season (summer or winter) and wood type (spruce, pine or mix), and potential interactions as fixed effects. Worker identity (ID) was treated as a random intercept, and company-specific residuals were estimated using restricted maximum likelihood (REML) with a variance component covariance structure. Model development involved forward selection of fixed effects, followed by likelihood ratio tests using maximum likelihood estimation to compare models. Sampling year as fixed effects and company-specific residuals did not contribute to the final models. Model fit was assessed through the Akaike Information Criterion (AIC) and inspection of standardized residuals, with the AIC guiding the selection of covariates. Final models were estimated using REML (see supplementary material, www.sjweh.fi/article/4240, file 1). From the regression coefficient and variance components, estimated arithmetic means (AM) of thoracic exposure levels were calculated for all combinations of company, job type, wood type and season (34). These group-level predictions were then assigned to workers for each of the two times when outcomes were measured (winters 2012/2013 and 2015/2016), excluding administrative staff, who were considered unexposed. Calculations and further details are provided in supplementary file 1.

### Blood sampling and analyses

Blood was collected using vacutainers without additives (BD Vacutainer, Franklin Lakes, NJ, US) and left at room temperature for 30–90 minutes. Serum was collected following 10 minutes of centrifugation at 3500 rpm. Samples for C-reactive protein (mCRP) and atopy testing were sent directly to a commercial laboratory for analysis, whereas the remaining serum samples were immediately frozen at -20 °C and transferred to -80°C upon arrival at the laboratory, where they were kept until analysis. Leucocytes, neutrophils, lymphocytes, monocytes and basophils in blood were counted at both study years 1 and 4, whereas erythrocytes and thrombocytes were counted at year 4 only. Multiplexed serum biomarker analysis was conducted on a Magpix instrument (BioRad, USA) using the Bio-Plex Manager MP Software. Analyte concentrations were obtained using Bio-Plex Data Pro software (BioRad, USA). The Human Magnetic Luminex Screening Assay LXSAHM-06 was used for IL-8, IL-1β, IL-10, IL-6, SP-D, TNF-α, whereas AHM-01 was used for CC-16, which is also called Uteroglobin (Bio-Techne, UK).

### Questionnaires

The participants completed a modified version of a published questionnaire suggested by the British Medical Research Council in 1960 (35), which focused on smoking and general health status. Additionally, they completed a separate questionnaire addressing work history and other characteristics (supplementary file 2).

### Data analysis

STATA/SE 18.0 software (StataCorp LP, Collage Station TX, USA) was used for statistical analysis which assessed longitudinal differences in serum markers between exposed and unexposed workers.

Estimated AM predictions of airborne exposure to thoracic dust, endotoxin, resin acid, monoterpenes, fungal spores and fungal fragments were assigned to each participant using the study JEM. As the exposure data were skewed and approximated log-normal distributions, ln-transformed values were used. Exposure levels were estimated from mixed models with person-ID as a random effect. An estimated geometric mean (GM) concentration with 95% confidence interval (CI) was calculated for each exposure component using the margins postestimation command in STATA. The geometric standard deviation for the GM_tot_ based on data from both years (GSD_tot_), as well as the GSD for between workers (GSD_bw_) and within workers (GSD_ww_) were calculated for each exposure component using the exponentiated square root of the random effect parameters of the models.

Serum concentrations of inflammatory biomarkers and blood cell counts, as well as associations between exposure and outcomes were estimated using mixed models with person-ID as a random effect, study year and exposure group as fixed effects, with adjustments for age, sex, body mass index (BMI) and current smoking. An interaction term of study year and exposure group was included in the models to assess whether changes in outcomes from baseline to follow-up differed between exposure groups. The estimated GM concentrations with 95% CI of serum biomarkers and blood cell counts were calculated from the mixed models by the margins postestimation command in STATA.

Any potential association between biomarker levels and the blood sampling time points (hour and minutes of the day) was tested by Pearson correlation analysis. A significant, small effect was observed for CC-16 (r_p_ -0.13, P<0.05). To check if this was linear and not diurnal, as might be expected, the association between time points and the log values of CC-16 were visually inspected and appeared to follow a linear trend over the time span of the measurements. To further assess this, we included a smooth spline function for time points in an additive model, which confirmed the linearity of the association. Mixed models of CC-16 were therefore adjusted for the time point (hour and minutes of the day) of blood sampling.

Assumptions of normal distribution, independence, and constant variance of the residuals, as well as linear relationship between explanatory variables and the outcomes were met. The assumption of constant variance of the residuals was checked visually by plotting residuals against fitted values.

## Results

A total of 450 exposed workers participated with blood samples, including 252 (56%) at both surveys, 124 (28%) only at baseline (2012/2013), and 74 (16%) only at follow-up (2015/2016). A total of 65 unexposed workers participated, including 37 (57%) at both surveys, 22 (34%) only at baseline, and 6 (9%) only at follow-up. The total numbers of observations were 702 for exposed and 102 for unexposed workers, with 376 and 59, respectively, at baseline and 326 and 43, respectively, at follow-up. An overview of the population characteristics is given in table 1. Exposed workers had higher proportion of men and were also more likely to smoke or to have smoked in the past. The exposed group also had a higher prevalence of asthma, but not chronic obstructive pulmonary disease (COPD).

**Table 1:** Characteristics of the study population

	Study year 1								Study year 4						
	Exposed workers (N=376)				Unexposed workers (N=59)				Exposed workers (N=326)				Unexposed workers (N=43)		
	N	Mean (min–max)	%		N	Mean (min–max)	%		N	Mean (min–max)	%		N	Mean (min–max)	%
Sex (male/female)	362/15		96/4		35/24		59/41		314/12		96/4		23/20		53/46
Never smokers	147		39		33		58		119		39		26		62
Current smokers	124		33		12		21		88		27		8		19
Ex-smokers	106		28		12		21		113		37		8		19
Age		46 (18–78)				51 (29–68)				47 (18–78)				53 (26–70)	
Body mass index		28 (19–45)				26 (20–37)				28 (19–45)				26 (21–35)	
Allergy (allergic rhinitis, atopic eczema)	92		24		12		21		89		27		10		23
Familiar asthma	99		26		14		24		88		27		8		19
Doctor-diagnosed asthma	44		12		3		5		39		12		2		5
Doctor-diagnosed COPD	6		2		1		2		5		2		0		0
Previous exposure to dust, fume or gases ^a^	289		77		21		36		247		76		16		37
Years of sawmillwork ^b^		18 (0–46)				3 (0–27)				19 (0–46)				3 (0–27)	
Any household or farm animal at home	219		59		35		60		7		41		1		33

^a^ Number of individuals previously exposed to any kind of dust, fume or gas at work. ^b^ Number of years working at a sawmill with wood dust exposure. Individuals tested twice: 253 exposed and 39 unexposed workers.

### Exposure

The exposed workers were assessed for exposure to dust, spores, resin acid, monoterpenes, endotoxins and fungal fragments as shown in table 2. The exposure did not differ significantly between study years. However, the variance was substantial and the differences between workers explained more of the total variance than the differences within workers, particularly for resin acids and monoterpenes.

**Table 2:** Exposure concentrations assessed at study year 1 (in 2012/2013), in study year 4 (in 2015/2016) and in both years summarized. [CI=confidence interval; GM=geometric mean of exposures; GSD_tot/bw/ww_=geometric standard deviation of the mean of exposures from both years combined/between workers/within workers]

Exposure parameter	Exposure assessments		Workers		Year 1			Year 4			Years 1 and 4 combined				
	N		N		GM_1_	95% CI		GM_4_	95% CI		GM_tot_	95% CI	GSD_tot_	GSD_bw_	GSD_ww_
Dust (mgm-3)	673		431		0.14	0.12–0.15		0.14	0.12–0.15		0.14	0.12–0.15	3.05	2.59	1.34
Spores (×103m-3)	673		431		42	38–48		45	40–50		43	39–48	3.33	2.71	1.57
Resin acids (µgm-3) ^a^	673		431		3.5	3.0–4.1		3.2	2.8–3.8		3.4	2.9–3.9	4.19	4.66	1.67
Monoterpenes (mgm-3) ^b^	673		431		1.6	1.3–1.9		1.6	1.3–1.9		1.6	1.3–1.9	4.98	7.70	1.70
Endotoxins (EUm-3)	673		431		2.4	2.2–2.6		2.4	2.2–2.6		2.4	2.2–2.6	2.65	2.15	1.20
Fungal fragments (×103m-3) ^c^	567		369		522	483–564		534	493–579		527	491–567	2.71	1.87	1.45

^a^ Sum of all measured resin acids (7-oxodehydroabietic acid, dehydroabietic acid, abietic acid, isopimaric acid and levopimaric acid). ^b^ Sum of all measured monoterpenes (α-pinene, β-pinene, limonene, 3-carene and p-cymene). ^c^ Sum of all sized fragments.

### Biomarker levels and associations with exposure

Small, but significant increases in serum CC-16 and mCRP were observed over the study period in the exposed group, whereas no significant changes in the unexposed group were observed (table 3). The changes in biomarker concentrations over the three years were not significantly different between exposed and unexposed workers. With data combined across study years, exposed workers had significantly lower serum-levels of IL-1β, TNF-α and IL-10 compared with unexposed workers (Table 3). Within the exposed group, the serum levels of mCRP increased by 70% (mean 1.11 mg/L, 95% CI 1.01–1.23) for each natural logarithmic unit increase (×2.7) of EU/m^3^ endotoxin (supplementary file 3, table a). Serum-levels of IL-10 increased by 75% (mean 1.07 pg/ml, 95% CI 1.02–1.12) for each increased log unit of mg/m^3^ monoterpene, TNF-α increased by 6.5% (mean 1.06 pg/ml, 95% CI 1.01–1.11) and IL-8 increased by 5% (mean 1.07 pg/ml, 95% CI 1.02–1.12), respectively, for each increased log unit of 10^3^ number of fungal spores/m^3^ (supplementary file 3, tables a–d). No other associations between exposure and biomarkers were observed.

**Table 3:** Serum concentrations of pro- and anti-inflammatory biomarkers. **Bold P-values indicate** significant differences between study years, or between exposed and unexposed groups in mixed models.

		Total N	Exposed									Unexposed									Change ^a^		Group ^b^
			N	Year 1			Year 4			Years		N	Year 1			Year 4			Years				
				Mean	95%CI		Mean	95%CI		P-value			Mean	95%CI		Mean	95%CI		P-value		P-value		P-value
Biomarkers ^c^																							
	IL-1-β (pg/ml)	720	632	13.4	11.7–15.3		14.0	12.3–16.0		0.134		88	21.3	15.4–29.5		23.0	16.7–31.8		0.260		0.652		**0.008**
	TNF-α (pg/ml)	767	673	15.6	14.0–17.5		15.8	14.1–17.64		0.472		94	22.3	17.3–28.8		22.5	17.5–29.1		0.844		0.371		**0.005**
	IL-6 (pg/ml)	609	531	2.3	2.0–2.5		2.3	2.1–2.6		0.285		78	2.8	2.1–3.7		2.7	2.0–3.6		0.688		0.432		0.203
	IL-8 (pg/ml)	768	672	20.6	18.6–22.7		21.4	19.4–23.6		0.091		96	23.8	18.7–30.4		25.6	20.1–32.5		0.160		0.533		0.264
	IL-10 (pg/ml)	541	471	1.4	1.2–1.6		1.4	1.2–1.6		0.974		70	1.9	1.5–2.6		1.9	1.4–2.6		0.822		0.823		**0.037**
	mCRP (mg/L)	789	688	1.5	1.4–1.6		1.7	1.5–1.8		**0.031**		101	1.6	1.3–2.1		1.7	1.3–2.2		0.690		0.686		0.568
	CC-16 (ng/ml) ^d^	774	678	15.9	15.2–16.5		16.7	16.0 –17.4		**<0.001**		96	16.9	15.1–18.9		16.9	15.1–19.0		0.989		0.184		0.314
	SP-D (ng/ml)	757	662	12.9	12.2–13.7		13.2	12.4–14.0		0.115		95	13.2	11.4–15.4		13.2	11.4–15.4		0.996		0.520		0.739

^a^ Differences in change from study year 1 to study year 4 between the groups (exposed versus unexposed). ^b^ Group differences (exposed versus unexposed) using data combined across study years. ^c^ All biomarkers are adjusted for age, sex, body mass index and current smoking. ^d^ CC-16 is adjusted for examination time.

### Differences in leucocyte counts between years and population groups

Higher counts of total leucocytes, neutrophils, lymphocytes and basophils and lower counts of monocytes and eosinophils were observed in year 4 compared to year 1 in exposed workers (table 4). Only the reduction in monocyte counts and the increase in basophil counts were significant in the unexposed group (table 4). The change in cell counts over the study years were significantly different between exposure groups for total leucocytes and basophils. With data across study years, there was no significant difference in blood cell counts between exposed and unexposed workers, nor any association with the concentration of exposure parameters (data not shown).

**Table 4:** Leucocyte counts. **Bold P-values indicate** significant differences between years (Diff. Year), or between exposed and unexposed groups in mixed models. Values are adjusted for body mass index, age, sex, and smoking. [CI=confidence interval; NA=not analysed, no blood sample this year]

		Total N	Exposed									Unexposed									Change ^a^		Group ^b^
			N	Study year 1			Study year 4			Diff. Year		N	Study year 1			Study year 4			Diff. Year				
				Mean	95% CI		Mean	95% CI		P-value			Mean	95% CI		Mean	95% CI		P-value		P-value		P-value
Cells (109/L)																							
	Erythrocytes ^c^	367	324	NA	NA		5.05	5.02–5.09		NA		43	NA	NA		5.04	4.93–5.16		NA		NA		0.910
	Leucocytes	789	688	7.12	6.93–7.30		7.59	7.40–7.78		**<0.001**		101	6.97	6.48–7.46		6.88	6.35–7.42		0.735		0.025		0.582
	Neutrophils	785	684	4.17	4.02–4.32		4.59	4.43–4.75		**<0.001**		101	4.16	3.76–4.57		4.18	3.73–4.63		0.944		0.085		0.970
	Lymphocytes	785	684	2.12	2.06–2.19		2.34	2.27–2.41		**<0.001**		101	1.97	1.80–2.15		2.10	1.92–2.29		0.076		0.276		0.118
	Monocytes	785	684	0.59	0.57–0.61		0.49	0.47–0.51		**<0.001**		101	0.59	0.54–0.64		0.45	0.39–0.50		**<0.001**		0.233		0.938
	Eosinophils	784	683	0.17	0.16–0.18		0.16	0.14–0.17		**0.012**		101	0.17	0.14–0.20		0.14	0.11–0.18		0.114		0.572		0.839
	Basophils	785	684	0.02	0.01–0.02		0.07	0.06–0.07		**<0.001**		101	0.02	0.01–0.03		0.04	0.03–0.06		**0.010**		**0.007**		0.708
	Thrombocytes ^c^	367	324	NA	NA		250	244–257		NA		43	NA	NA		233	213–253		NA		NA		0.11

^a^ Differences in change from study year 1 to study year 4 between the groups (exposed vs unexposed); ^b^ Group differences (exposed vs unexposed) using data combined across study years. ^c^ Thrombocytes and erythrocytes were counted only in year 4.

### Associations between leucocytes and biomarkers

**Figure 1:**
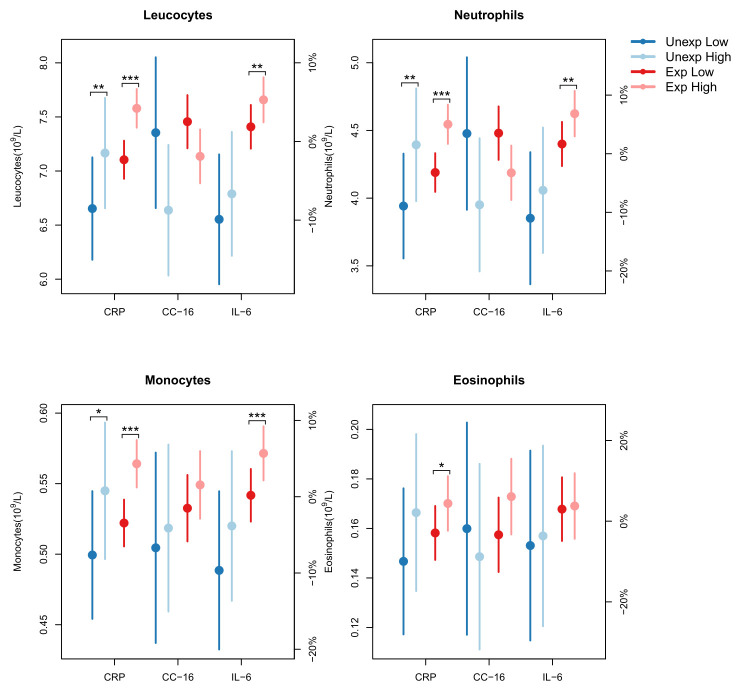
Associations between cell counts and biomarker concentrations in blood of sawmill workers. Mixed-model estimates (with 95% confidence intervals) of the effect of a 1-unit increase in the natural log-transformed levels of the biomarkers mCRP, CC-16, and IL-6 on leukocyte, neutrophil, monocyte, and eosinophil counts. The model was adjusted for sex, exposure, current smoking, age, BMI, and study year, and included an interaction between exposure and study year. For CC-16, an additional adjustment was made for time of examination. Cell counts were evaluated at the mean log-biomarker level ± 0.5 for each biomarker. The percentages on the right y-axis were computed with respect to the mean cell count.

Leucocyte counts were not associated with the lower levels of cytokines observed in the exposed group. However, several of the elevated leucocyte counts were associated with concurrent increased mCRP or IL-6 concentration. For total leucocytes the mean increase was 6% per log-unit increase (2.7 mg/L) of mCRP (figure 1). Neutrophils showed a mean increase of 8.4%, while monocytes and eosinophils each increased by 8%. This trend was predominantly observed in the exposed group. Similarly, leucocytes, neutrophils and monocytes increased with rising IL-6 concentrations. Leucocytes increased by 3.3% per log-unit increase (2.7 pg/ml) of IL-6, with the increase being 11% higher in exposed workers compared to unexposed workers over the three-year study period. Neutrophils increased by 5%, with the exposed group showing a significantly greater increase of 14%. For monocytes the increase was 5.4%, mainly driven by the exposed group. Conversely, elevated levels of CC-16 were associated with reductions in leucocyte and neutrophil counts. Total leucocytes decreased by 7% and neutrophils by 9.6%, per log unit increase of CC-16 (figure 1). This reduction was most pronounced in the unexposed workers (supplementary file 3, tables e–m).

## Discussion

This was a longitudinal study with unbalanced repeated measurements that explored associations between exposure to wood dust and related resin acids, microbial or volatile components and inflammatory markers in sawmill workers over a three-year period. Changes in inflammation-related biomarkers differed between exposed and unexposed workers, suggesting chronic immune modulation in response to workplace conditions.

The stability of exposure levels across the three years of the study reflects consistent work practices that maintain a steady exposure environment. During the three years, exposed workers showed increases in total leucocyte counts and the inflammatory markers serum CC-16 and mCRP; these changes may be indicators of ongoing inflammatory responses (29, 36). Exposed workers showed overall lower serum levels of IL-1β, TNF-α and IL-10 compared to unexposed workers, suggesting an immune shift towards an attenuated but persistent, low-grade inflammatory state. Stable levels of other markers like IL-6, IL-8 and SP-D indicated that the immune response might be balanced towards a more muted inflammatory response. Overall lower serum levels of IL-β, TNF-α and IL-10 in the exposed group, with mCRP levels remaining relatively high, suggest a low-grade chronic inflammatory condition with insufficient anti-inflammatory signaling, which may reflect an adaptation response, where immune responses are dampened due to chronic occupational exposure and associated inflammation.

Previous studies in different wood-processing contexts show mixed results regarding cytokine changes. A cross-sectional study from Ghana reported that except for IL-2, the expression of all serum cytokines, including IL-10, was higher in woodworkers compared to a nonexposed control group (37). Priha et al (38) reported a higher cross-shift reduction of IL-6, IL-8 and TNF-α in nasal lavage of factory workers working with birch and pine than those working with medium density fiberboard (MDF) and unexposed controls, respectively. All workers also had higher symptom frequencies than controls, but the wood workers also reported more eye- and throat symptoms than the MDF workers.

Several studies have confirmed that IL-1β and TNF-α play a central role in the development of lung inflammation induced by exposure to organic dust, mineral fibers and fungal spores (18, 39). The reduced serum level of IL-1β, TNF-α and IL-10 in the exposed workers in the present study is different from what others have found in mouse or *in vitro* studies, where an increase in these cytokines has been related to (short-term) wood dust exposure (18). However, more consistent with our findings, a Finnish *in vitro* study, found that wood dust exposure of mouse macrophages caused a dose-dependent inhibition of IL-1β mRNA expression (40). Although the exact mechanisms were unclear, as no inhibition was observed after exposure to TiO_2_ in comparison, it is likely to be caused by wood-dust derived components. Terpenes are known to reduce the expression of TNF-α, IL-1β and IL-6 in RAW 264.7 macrophages as well as in several *in vivo* animal models, with suspected inhibition of NF-KB signaling pathway, although involvement of other pathways could not be excluded (15). Natural terpenes released from the wood may be possible inhibitors of inflammation, although no such association was shown in our study. Long et al (41) reported that induction of TNF-α and MIP-2/CXCL2 expression and protein release in rat alveolar macrophages after pine dust exposure is partly mediated by reactive oxygen species. TNF-α plays an important role as a mediator of the respiratory tract’s response to particles. Furthermore, resin acids in wood dust can be a contributing factor as several resin acids have been shown to produce lytic damage to alveolar, tracheal, and bronchial epithelial cells (16). Wood dust can indeed induce oxidative stress in wood workers, as shown by Ghelli et al (42). An association of wood dust exposure with elevated indices of inflammation, oxidative stress, lipid peroxidation, oxidative DNA damage and reduction in antioxidants and peak expiratory flow rate were shown among South-Nigerian wood workers (43). Furthermore, several terpenes found in wood have anti-oxidative effects (15) and may also in this regard have protective effects. Although terpenes have properties that may reduce inflammation, inhalation of concentrations above the occupational exposure limit of 140 mg/m^3^ (44) may result in irritation of the airways.

The relationship between exposure and serum-levels of cytokines was evident at the group level, with clear differences observed between exposed and unexposed workers. Although group differences may be evident in occupational exposure studies, they do not always reflect corresponding dose-response or linear relationships (45, 46). This may occur due to variability in individual susceptibility, exposure misclassification or non-linear kinetics. Nevertheless, some cytokine levels in the current study showed modest correlations with specific quantitative exposure measurements. Higher monoterpene exposure was associated with increased IL-10, suggesting an anti-inflammatory response (31, 32). An exposure-induced inflammatory response may be followed by a natural anti-inflammatory response to maintain homeostasis. Increased levels of CC-16 in the exposed group support this counter-response, although no association with the exposure levels were found. A similar increase in serum CC-16 have previously been observed among grain dust-exposed workers, without any association with exposure measurements or respiratory health effects (45). This may indicate that increased serum CC-16 in the present study is a reversible defense reaction rather than early sign of lung damage. CC-16 have been shown to have a protective role in asthma, being diminished in individuals with low lung function and frequent asthma symptoms (47). On the other hand, TNF-α increased slightly with higher spore exposure, suggesting a fungal-associated immune activation (24). The slight increase in IL-8 with increasing fungal spore exposure suggests that this exposure triggered a localized inflammatory response, involving the recruitment and activation of neutrophils (25, 26). Endotoxin exposure was strongly and positively associated with serum mCRP, further linking endotoxin exposure with systemic inflammation. Although not uniformly strong, these associations highlight complex immune adjustments to specific wood dust components and support the concept of a muted immune response at low, but persistent exposure levels.

Higher leucocyte counts in exposed workers indicate an immune response potentially triggered by inhaled dust particles, with increased neutrophils suggesting localized inflammatory responses. The association between increased leucocyte counts and concurrent increased mCRP and IL-6 supports this, whereas the anti-inflammatory role of CC-16 is reflected by lower leucocyte counts with increased CC-16 levels. Similar changes in cell counts were reported for wood workers processing tropical hardwood in Ghana (37), and in bronchoalveolar lavage (BAL) fluid after exposure of healthy volunteers to wood chip mulch dust (48). Reduced monocytes and eosinophils in exposed workers, highlight a nuanced immune shift, possibly due to feedback mechanisms responding to chronic exposure and the lower systemic levels of IL1-β, TNF-α and IL-10 (25, 49, 50). While eosinophils are typically involved in allergic reactions and parasitic infections, the small difference between year 1 and 4 suggests that wood dust exposure is not causing an allergic response in the sawmill workers. In contrast, in another study among healthy volunteers from the general population, increased numbers of T-lymphocytes and eosinophils in BAL fluid were observed after exposure to pine dust (51). These differences may be due to different wood having differential effects on inflammatory responses, as shown in mouse-models with dominant infiltration of eosinophils resulting from birch exposure, while neutrophils and lymphocytes dominated after oak exposure (18). Another reason may be the difference in exposure duration ie, acute versus long-term exposure.

A key strength of the present study is the large sample size and the detailed exposure assessment that enhanced our ability to identify exposure contrast and accordingly to detect associations with inflammatory marker levels in serum. The rigorous exposure measurements and a thorough understanding of the participating sawmills likely minimized exposure misclassification. Furthermore, the longitudinal design, paired with comprehensive assessment of various inflammatory markers and immunologically relevant cell counts, provided valuable insights into changes over a three-year period. However, longer studies may be needed to further understand the long-term impacts of exposure on immune and inflammatory markers and associated health outcomes. There may also have been potential confounding factors that were not accounted for in the current investigation, such as environmental exposures or lifestyle characteristics including diet, physical activity, and stress levels, which could have influenced biomarkers or white blood cells counts. However, we would expect these factors to be about equally distributed in both exposed and unexposed workers, so unlikely to explain any differences observed between the two groups.

Despite a comprehensive and detailed exposure assessment, the association of biomarker changes with exposure may not have been entirely elucidated. Small effects of measured exposure components such as fungal spores and resin acids were observed, but the strongest differences were between the exposed and the unexposed group. This likely reflects multiple and complex factors related to relevant work exposures and a likewise complex response system of cells and signaling pathways that may result in respiratory symptoms.

### Concluding remarks

This study showed that continuous exposure to wood dust and related components over three years may result in specific immune changes, with a shift in cytokine profiles toward lower pro-inflammatory markers and chronic low-grade inflammation. A positive association was found between predicted exposure levels of endotoxin and increased mCRP, monoterpenes and increased IL-10, and fungal spores and TNF-α and IL-8, indicating exposure-induced inflammation. The exposed workers showed an increase in mCRP and CC-16 over three years, not seen in the unexposed workers. This was supported by a positive association of mCRP and IL-6 with elevated leukocyte counts in exposed workers, while elevated CC-16 levels were associated with reduced neutrophils in unexposed workers. However, the immune system in exposed workers showed mixed cytokine changes, with notably lower serum levels of IL1-β, TNF-α and IL-10, suggesting a shift towards a less regulated, potentially more muted immune state. These results highlight the necessity for effective protective measures and long-term monitoring strategies to better understand the mechanisms underlying these immune changes and mitigate the adverse health effects of wood dust exposure in sawmill workers.

### Disclaimer

The findings and conclusions in this article are those of the authors and do not necessary represent the official position of the National Institute for Occupational Safety and Health, Centers for Disease Control and Prevention.

## Supplementary material

Supplementary File 1

Supplementary File 2

Supplementary File 3
