# Supplementary material for: Immune modulating effects of continuous bioaerosol and terpene exposure over three years among sawmill workers in Norway
Source: Scand J Work Environ Health. 2025 Aug 29;51(5):433–43. doi: 10.5271/sjweh.4240 (PMC12415592; doi:10.5271/sjweh.4240)
Supplement: Supplementary File 2 [file SJWEH-51-433-S002.pdf]

Statens arbeidsmiljøinstitutt | National Institute of Occupational Health

ID no.:…………………………………………………………… Date:…………………………………..

# Questions about current and previous job

1. Current employer/sawmill:……………………………………………………………………………………
2. Employed since (month/year):…………………………………………
3. Current job title:…………………………………………………………………………………………………….
4. Which tasks do you have with this job title?.........................................................
5. When did you start working with this job title? (month/year)……………………………………………..
6. In which department do you work?.......................................................................
7. Do you work full time or part time?

   Full time ☐ part time ☐ Employment- %:………………………………….

8. Do you have work shifts? No ☐ Yes ☐

   If yes, what kind of shift do you work?……………………………………………………………………………

9. Do Do you wear gloves or respiratory protective equipment at work?

   No ☐

   Yes, gloves ☐ Form/type?:………………………………………………………………………

   Yes, resp.prot.equip. ☐ Type:…………………………………………………………………………………

   If yes, what, how often, and if it is only at spesific job tasks:

| | Gloves | Respirator | At specific job tasks: |
|---|---|---|---|
| Daily | ☐ | ☐ | ………………………………………………………………………… |
| Weekly | ☐ | ☐ | ………………………………………………………………………… |
| Few times a year | ☐ | ☐ | ………………………………………………………………………… |

10. Have you previously worked in other departments in this sawmill?

No ☐ Yes ☐ If yes, please fill in:

| | Department | Job title | Start (mnth/yr) | End (mnth/yr) |
|---|---|---|---|---|
| 1 | | | | |
| 2 | | | | |
| 3 | | | | |
| 4 | | | | |

11. Other jobs before you started working in the sawmill?

No ☐ Yes ☐

If yes, please fill in:

| | Company name/Company type | Work tasks | Start (mnth/yr) | End (mnth/yr) |
|---|---|---|---|---|
| 1 | | | | |
| 2 | | | | |
| 3 | | | | |
| 4 | | | | |

12. Were you exposed to fumes, dust or irritating vapors (gasses)on a regular basis during your previous work before you started working in the sawmill?

No ☐

Yes ☐ If yes, which exposure:

Asbestos ☐ Fibers (ceramic/minerals) ☐

Solvents ☐ Welding/cutting ☐

Quartz/sand blasting ☐ Gas ($SO_2$, fluor, etc.) ☐

Organic dusts (animals, farm) ☐ DiIsocyanates, hardener, plastic, etc. ☐

Woddust ☐ Oildust ☐

Other ☐ Specify:…………………………………………………………………….

ID no.:…………………………………………  Date:……………………………………

# Airway symptoms and health, first time examination

(The follow-up scheme should also be filled in by the first time examination)

| | Yes | No | Don't know |
|---|---|---|---|
| 1. Have you ever suffered from allergic rhinitis? | ☐ | ☐ | ☐ |
| 2. Did you suffer from allergic eczema (atopic eczema) as a child? | ☐ | ☐ | ☐ |
| 3. Have any of your parents, grandparents, brothers or sisters ever had asthma or asthmatic bronchitis? | ☐ | ☐ | ☐ |

4. Before you started in your present job, have you ever been diagnosed by a doctor with asthma or asthmatic bronchitis?

| | Yes | No | Don't know |
|---|---|---|---|
| a. Yes, in childhood | ☐ | ☐ | ☐ |
| b. Yes, in adulthood | ☐ | ☐ | ☐ |

5. Have you ever been diagnosed with any of the following chronic diseases by a doctor?

| | Yes | No |
|---|---|---|
| a. Chronic bronchitis | ☐ | ☐ |
| b. Chronic obstructive pulmonary disease (COPD) | ☐ | ☐ |
| c. Cardiovascular diseases (AMI, angina, hypertention etc.) | ☐ | ☐ |
| d. Other chronic disease? …………………………………………………… | | |

6. Are you sensitive or allergic to any of the following? If yes, what sort of symptoms do you have?

| | Please, specify | Yes | No | Don't Know | Symptoms | | | |
|---|---|---|---|---|---|---|---|---|
| | | | | | Sneezing or running nose | Chest thightnes and wheezing | Itching or red skin | Itching or watering eyes |
| Wooddust | | | | | | | | |
| Molds | | | | | | | | |
| Housedust | | | | | | | | |
| Food | | | | | | | | |
| Animals | | | | | | | | |
| Plants, grasses | | | | | | | | |
| Other | | | | | | | | |
| Other | | | | | | | | |

7. Did you grow up on a farm? Yes ☐ No ☐

8. Do you smoke or have you ever smoked more than one cigarett a day?

   a. No, never smoked ☐

   b. Yes, but stopped more smoking more than 1 year ago ☐

   c. Yes, but stopped smoking less than 1 year ago ☐

   d. Yes, I smoke ☐

   e. Yes, I smoke but not daily (”Partysmoker”) ☐

9. For how many years have you smoked/did you smoke? ………………… years

10. In the time you have been smoking, aproximately how many cigarettes have you smoked a day?
    (one packet of tobacco is equivalent to 50 cigarettes)

    a. 1 – 9 cigarettes a day ☐

    b. 10 – 19 cigarettes a day ☐

    c. 20 cigarettes or more a day ☐

11. At what age did you start smoking? ………………….. years

| | Yes | No | Don’t know |
|---|---|---|---|
| 12. Are you exposed to secondhand smoke on a daily basis? | ☐ | ☐ | ☐ |
| 13. Were you exposed to secondhand smoke as a child? | ☐ | ☐ | ☐ |

14. Any comments?

……………………………………………………………………………………………………………………

……………………………………………………………………………………………………………………

……………………………………………………………………………………………………………………
